# A Case of Junctional Ectopic Tachycardia and Complete Atrioventricular Block After Transcatheter Aortic Valve Replacement

**DOI:** 10.1111/anec.70122

**Published:** 2025-11-05

**Authors:** Shingo Yoshimura, Suguru Ueba, Kenichi Kaseno, Kohki Nakamura, Shigeto Naito

**Affiliations:** ^1^ Division of Cardiology Gunma Prefectural Cardiovascular Center Maebashi Gunma Japan

**Keywords:** atrioventricular block, junctional ectopic tachycardia, narrow QRS tachycardia, pacemaker implantation, transcatheter aortic valve replacement

## Abstract

Junctional ectopic tachycardia (JET), a tachyarrhythmia originating from the atrioventricular (AV) node and/or bundle of His, is commonly observed in pediatric patients following congenital heart surgery. JET is characterized by a heart rate above the 95th percentile for age, whereas rates below this threshold are referred to as accelerated junctional rhythm (AJR). Although AJR with a potential risk of developing AV block has been reported following transcatheter aortic valve replacement (TAVR), no cases of JET following TAVR have been documented. We report a case of JET and complete AV block observed after TAVR, which was effectively managed with medication and permanent pacemaker implantation.

## Graphical abstract

This case highlights junctional ectopic tachycardia (JET) and complete atrioventricular (AV) block after transcatheter aortic valve replacement (TAVR). JET after TAVR may be associated with AV block, potentially requiring rate control with medication and pacemaker implantation.

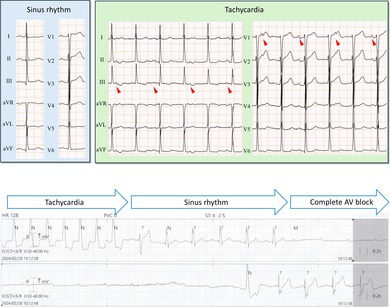

An 89‐year‐old woman underwent transcatheter aortic valve replacement (TAVR) for severe aortic stenosis. A 23 mm Navitor valve (Abbott Structural Heart, Santa Clara, CA, USA), a self‐expanding device, was implanted via the transfemoral approach (Figure 1A,B). No atrioventricular (AV) block or junctional rhythm was observed during the procedure. Electrocardiogram (ECG) immediately after TAVR showed sinus rhythm with no conduction system disturbances (Figure 2A). On postoperative Day 2, ECG revealed a regular, narrow QRS tachycardia with a cycle length of 600–640 ms and QRS morphology identical to sinus rhythm (Figure 2B). During tachycardia, ventriculoarterial (VA) dissociation and intermittent right or left bundle branch block (RBBB or LBBB) were observed (Figure 2C). The cycle length of the tachycardia gradually shortened, and by postoperative Day 5, the tachycardia persisted with a cycle length of 470–490 ms, accompanied by LBBB (Figure 2D).

**FIGURE 1:**
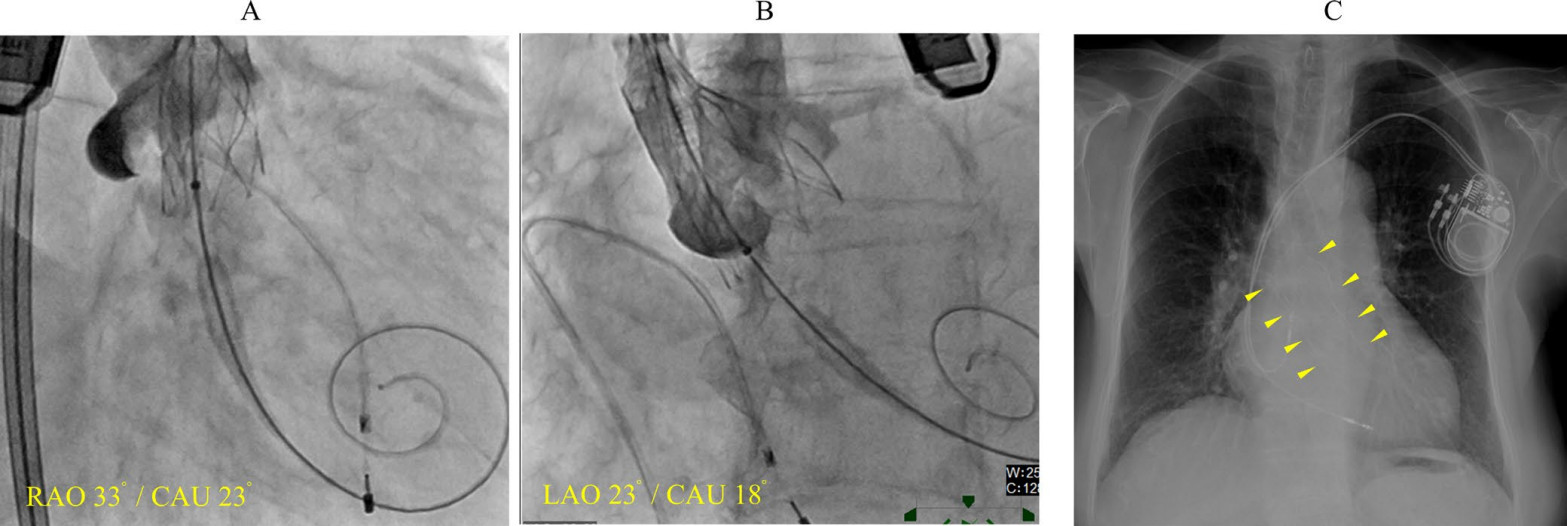
(A, B) Angiography during transcatheter aortic valve replacement. (A) Cusp‐overlap view. (B) Three‐cusp view. (C) Postoperative chest X‐ray following permanent pacemaker implantation. Arrows indicate the prosthetic aortic valve. CAU, caudal; LAO, left anterior oblique; RAO, right anterior oblique.

**FIGURE 2:**
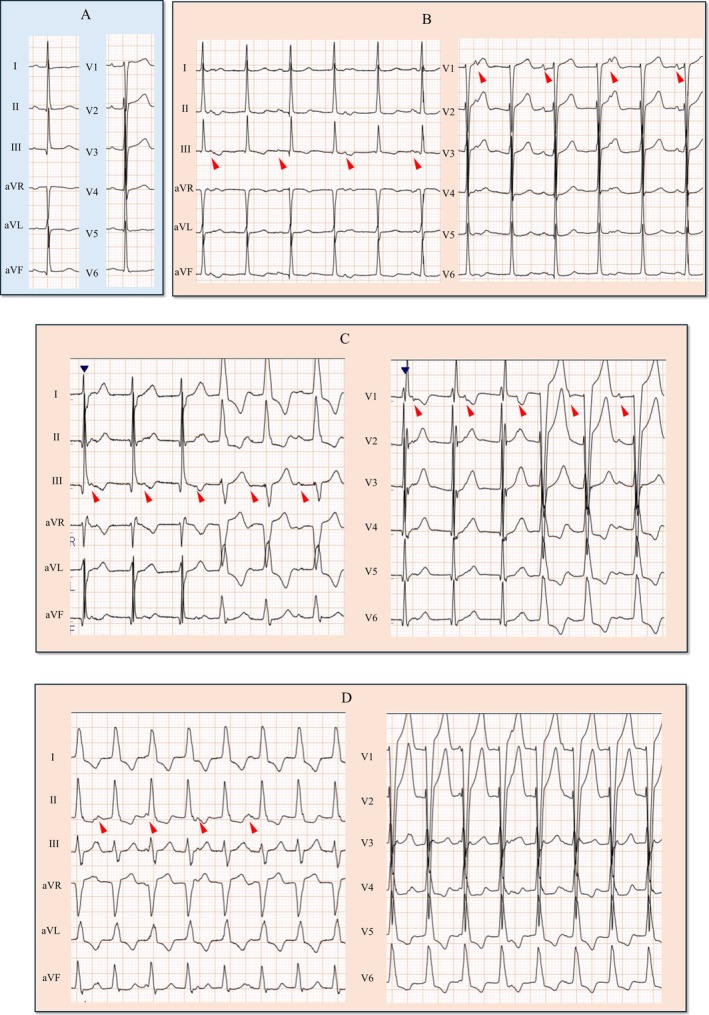
Twelve‐lead electrocardiogram (paper speed 25 mm/s). (A) Sinus rhythm immediately after transcatheter aortic valve replacement (TAVR). (B) Narrow QRS tachycardia with a cycle length of 600–640 ms on postoperative Day 2 following TAVR. (C) Tachycardia with intermittent right and left bundle branch block patterns. (D) Tachycardia with a shortened cycle length (470–490 ms) accompanied by a left bundle branch block pattern on postoperative Day 5 following TAVR. Arrows indicate P waves with ventriculoarterial dissociation during tachycardia.

On postoperative Day 6, the patient experienced syncope while lying in bed during the daytime, without identifiable prodromal symptoms. ECG revealed a transient complete AV block following the restoration of sinus rhythm, resulting in cardiac arrest (Figure 3). Consequently, a permanent pacemaker was implanted (Figure 1C).

**FIGURE 3:**
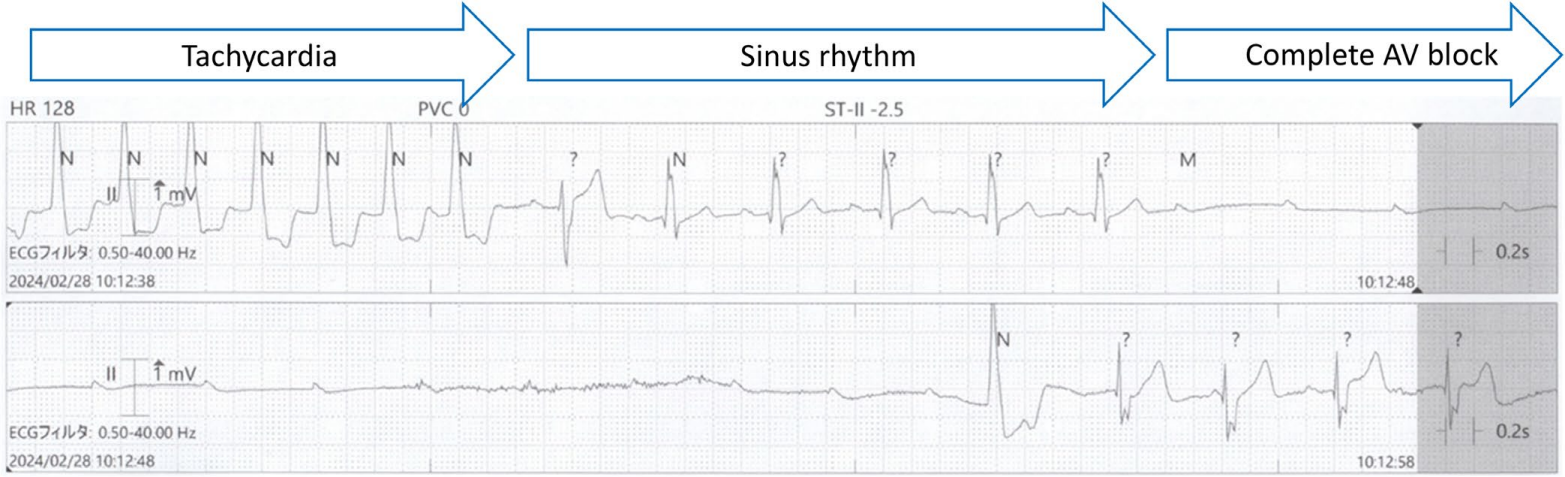
Electrocardiogram (lead II) on postoperative Day 6 showing tachycardia with a left bundle branch block pattern, followed by a return to sinus rhythm and subsequent progression to complete atrioventricular block.

After pacemaker implantation (PMI), manual atrial and ventricular pacing was performed via the pacemaker. Tachycardia was not suppressed by atrial pacing at a cycle length of 460 ms but was temporarily terminated at 440 ms, although it recurred within seconds (Figure 4). Similarly, tachycardia was transiently suppressed by ventricular pacing at cycle lengths of 420 ms or below; however, no pacing protocol was able to definitively terminate the tachycardia.

**FIGURE 4:**
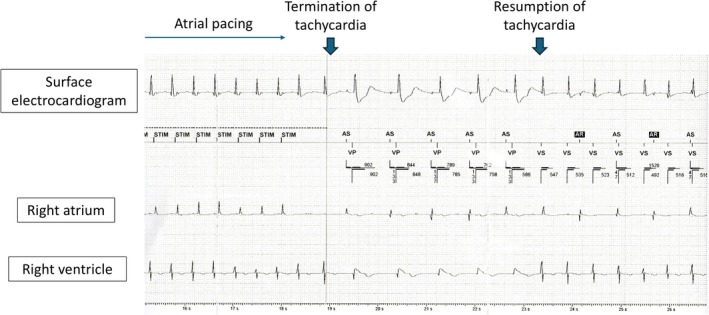
Intracardiac electrogram from the pacemaker. The electrogram shows tachycardia terminated by atrial pacing with a cycle length of 440 ms; however, it resumed after five beats.

Because tachycardia persisted, the patient was administered a β‐blocker (bisoprolol 5 mg/day) and a calcium channel blocker (diltiazem 100 mg/day). Although the tachycardia did not entirely resolve, medication effectively reduced the patient's heart rate to below 100 bpm, and she was discharged on postoperative Day 15.

At the 1‐month follow‐up, pacemaker interrogation revealed that the tachycardia had resolved, with a return to either sinus rhythm or atrial pacing and a ventricular pacing burden of < 1%.

Differential diagnoses for narrow QRS tachycardia include supraventricular tachycardia, Junctional ectopic tachycardia (JET) and upper septal ventricular tachycardia (VT). VA dissociation during tachycardia ruled out atrial tachycardia and AV reciprocating tachycardia, which require atrial involvement for the reentry circuit. Additionally, ventricular pacing by the pacemaker during sinus rhythm showed no VA conduction, making AV nodal reentrant tachycardia unlikely. Alternating right and left BBB patterns further suggested that upper septal VT was unlikely. Given that the QRS morphology during tachycardia mirrored sinus rhythm, we concluded that the tachycardia originated from the upper regions of the His‐Purkinje conduction system, supporting a diagnosis of JET (Collins et al. 2009).

In a study of 301 patients who underwent TAVR, accelerated junctional rhythm (AJR) was observed in six patients on the day of the procedure, of whom two (33%) progressed to complete AV block and required PMI (Angsubhakorn et al. 2020). In these patients, the mean heart rate during AJR was 68.2 ± 12.1 bpm, and all cases returned to sinus rhythm within 2 days. Unlike AJR in these patients, JET after TAVR in our case had a heart rate exceeding 100 bpm, persisted for an extended duration, and required treatment. To our knowledge, this has not been previously reported in the literature. However, the study suggests a shared pathology between AJR and AV block, similar to the relationship observed between JET and AV block in our case. Therefore, the presence of JET after TAVR warrants careful monitoring for potential AV block.

The presence of alternating BBB also suggested the potential for progression to complete AV block. One possible mechanism is simultaneous conduction block of the right and left bundle branches. However, if this had occurred, we would have expected to observe ventricular asystole even during tachycardia. Because ventricular asystole was noted only after the termination of tachycardia, it remains possible that complete AV block occurred at a level superior to the bundle branches and the origin of JET.

The complete AV block was transient, and no conduction abnormalities were observed at the 1‐month follow‐up, raising questions about the necessity of permanent PMI. Paroxysmal AV block is classified into two types based on its mechanism: intrinsic AV block and vagally mediated AV block. The former requires PMI, whereas the latter does not. In this case, AV block occurred during the daytime without identifiable triggering factors, and BBB was observed, making vagally mediated AV block unlikely. Additionally, vagally mediated AV block is typically associated with sinus rate slowing; however, no prolongation of the PP interval was observed during ventricular asystole. Based on these findings, we diagnosed the AV block as intrinsic.

Since JET in this case required rate control with medication, which could potentially exacerbate conduction disturbances, pacing support was necessary. Although a temporary external pacemaker could have been considered to allow time for conduction system recovery, previous studies have shown that complete AV block occurring more than 24 h after TAVR is associated with an increased risk of persistent AV block (Kagase et al. 2021). As the AV block occurred on postoperative Day 6, we opted for permanent PMI. A leadless pacemaker could have been a less invasive alternative; however, a transvenous dual‐chamber pacemaker was chosen to prevent medication‐induced sinus bradycardia and maintain AV synchrony, which is critical for preventing heart failure. However, further research is needed to determine whether permanent PMI is essential in similar cases.

The pathogenesis of JET remains unclear, but according to previous studies, postoperative JET may be triggered by mechanical trauma or inflammation at the AV junction (Kusterer et al. 2018). In cases of AV block following TAVR, similar factors (e.g., mechanical trauma from the valve prostheses, local inflammation, edema, and myocardial ischemia) are implicated (Lee et al. 2018). In the present case, considering the resolution of JET and conduction disturbances at the 1‐month follow‐up, transient inflammation, edema, and myocardial ischemia post‐TAVR likely contributed to both JET and complete AV block. Additionally, the heart rate of the tachycardia gradually increased and was transiently suppressed by overdrive pacing, suggesting that enhanced automaticity—representing a progression from AJR within a continuous process—could be a plausible mechanism.

## Author Contributions

Shingo Yoshimura and Suguru Ueba contributed to manuscript preparation, data collection, and data interpretation. All other authors contributed to manuscript revision and approved the final version of the manuscript.

## Ethics Statement

The authors have nothing to report.

## Consent

Informed consent was obtained from the patient.

## Conflicts of Interest

The authors declare no conflicts of interest.

## Data Availability

The data that support the findings of this study are available from the corresponding author upon reasonable request.
